# Emergence of microbial networks as response to hostile environments

**DOI:** 10.3389/fmicb.2014.00407

**Published:** 2014-08-19

**Authors:** Dario Madeo, Luis R. Comolli, Chiara Mocenni

**Affiliations:** ^1^Department of Information Engineering and Mathematics, University of SienaSiena, Italy; ^2^Structural Biology and Imaging Department, Life Sciences Division, Lawrence Berkeley National LaboratoryBerkeley, CA, USA

**Keywords:** microbial communities, bacterial social networks, evolutionary games, graph theory, evolutive decisions, hostile environmental conditions

## Abstract

The majority of microorganisms live in complex communities under varying conditions. One pivotal question in evolutionary biology is the emergence of cooperative traits and their sustainment in altered environments or in the presence of free-riders. Co-occurrence patterns in the spatial distribution of biofilms can help define species' identities, and systems biology tools are revealing networks of interacting microorganisms. However, networks of inter-dependencies involving micro-organisms in the planktonic phase may be just as important, with the added complexity that they are not bounded in space. An integrated approach linking imaging, “Omics” and modeling has the potential to enable new hypothesis and working models. In order to understand how cooperation can emerge and be maintained without abilities like memory or recognition we use evolutionary game theory as the natural framework to model cell-cell interactions arising from evolutive decisions. We consider a finite population distributed in a spatial domain (biofilm), and divided into two interacting classes with different traits. This interaction can be weighted by distance, and produces physical connections between two elements allowing them to exchange finite amounts of energy and matter. Available strategies to each individual of one class in the population are the propensities or “willingness” to connect any individual of the other class. Following evolutionary game theory, we propose a mathematical model which explains the patterns of connections which emerge when individuals are able to find connection strategies that asymptotically optimize their fitness. The process explains the formation of a network for efficiently exchanging energy and matter among individuals and thus ensuring their survival in hostile environments.

## 1. Introduction

How spatial and temporal organization in cell-cell interactions are achieved remains largely elusive. The central hypothesis of this unique topic is that a broad range of interactions and connections forming networks across species define strategies for co-evolution, and the display of adaptive strategies from microbial networks as a response to altered environments (Ben-Jacob and Cohen, 1997; Ben-Jacob et al., 2000; Dwyer et al., 2008). These are questions of great interest in a rapidly evolving area of science.

Surfaces concentrate nutrients, and it is generally assumed that planktonic cells were the first to take advantage of the catalytic and protective advantages offered by surfaces—a first step in the development of biofilms. Likely the first complex systems to achieve homeostasis in response to fluctuations in the primitive Earth environment, biofilms facilitated the development of complex interactions between individual cells and the development of signaling pathways and chemotactic motility (Hall-Stoodley et al., 2004; Fuhrman, 2009; Gure, 2009; Shimoyama et al., 2009). Interactions between two species of microbes that affect their coexistence and evolution have been studied in a laboratory system of two species (Hansen et al., 2009). Direct cell-cell interspecies interactions have been reported for laboratory cultures (Dubey and Ben-Yehuda, 2011) and for intact microbes in natural communities (Comolli and Banfield, 2014).

New metagenomics data from environmental microbial communities (e.g., Wrighton et al., 2012; Kantor et al., 2013) is showing that novel, small microorganisms lack the full metabolic potential to have a truly independent lifestyle. In other work (Baker et al., 2010; Comolli and Banfield, 2014) linking genomics and imaging, one novel nanoarchaea named ARMAN has been found establishing connections with archaea of different species. Therefore, we start with microbes of different classes or species assuming they lack the full metabolic potential necessary for survival under certain conditions; we also assume the two species complement the needs of each other, so that individual of one species seek the complement provided by individuals of the other species.

Evolutionary game theory (Weibull, 1995; Hofbauer and Sigmund, 1998; Nowak, 2006b) provides a rather intuitive framework to model interactions and decisions among co-evolving members of a population. It has been successfully used to describe relevant mechanisms of cooperation in biology (Nowak, 2006a; Frey, 2010) as the result of collective behavior induced by altruistic decisions, which allows a population to increase its own fitness.

The present study is an attempt to model the activation of connections among the members of a population of microorganisms accounting for the new findings on microbial communities formation. Starting from evolutionary game theory we are interested in understanding the conditions (for the simplified variables and parameters of our model) allowing the onset of cooperative interaction between two or more individuals, and their sustainment in the most efficient networks. Specifically the formation of suitable physical connections will eventually allow them to exchange a certain amount of energy and matter. The activation of links among microorganisms gives rise to the formation of a network, which increases the probability that any single individual and the whole population will survive.

The proposed approach is based on a recent paper (Madeo and Mocenni, under revision) where evolutionary game theory is extended to model the behavior of a finite population in which members are organized according to a network of relationships between them, such as friendship, spatial proximity, sharing, etc. The model introduced in that paper describes decisions of single individuals rather than average strategies of the whole population, and provides a natural tool to deal with the problem of connection formation among couples of bacteria from a single-cell fitness perspective—we will use the term “bacteria” for simplicity, but they could also be archaea or protists. The main idea motivating the present paper is that two microorganisms establish a connection when it provides a significant gain to both. This paradigm assumes that two classes of bacteria with different biological characteristics are present in the system, and under averse environmental conditions members of one class may need to access resources that only the individuals of the other class are able to produce, and viceversa.

In terms of game theory the propensity of a bacterium to establish a connection with another one is a behavioral strategy, namely a *game strategy*. Each strategy provides a certain payoff which accounts for natural constraints, such as distance and available energy. A connection strategy becomes effective if both involved individuals take an enough great advantage from it. This process can be described as a game where available strategies to the players are decisions to connect. Evolutionary games and the replicator equations introduced in Madeo and Mocenni (under revision) provide the mathematical models allowing us to follow the system dynamics of connections of a single bacterium and network formation at a global level. As a result, if the environmental conditions are favorable, the bacteria spend their energy only on surviving and reproducing. To the contrary, under averse conditions they acquire/transfer a certain amount of available energy from/to the connected bacteria. A similar phenomenon is the well known mechanism of coordinated motion and aggregation shown by the starving amoeba *Dictyostelium discoideum* (Eichinger and Noegel, 2003). Moreover, we assume that the bacteria are not allowed to move. Indeed, in the present work we are mainly focused on introducing a methodology that will be leverage in future works, and where the models will be compared with experimental data.

The approach proposed in this paper implicitly incorporates mechanisms of cell-cell recognition underlying interspecies interactions, and uses theory and modeling to show the emergence of counterintuitive patterns resulting, for example, from long term communication mechanisms. Our results show that the formed networks optimize the use of the available energy produced and exchanged between microorganisms. We also find conditions or regions of the parameter space which clearly enable or prevent efficient outcomes.

The paper is organized as follows: Section 2 describes the components of the mathematical model, such as bacteria, decisions and payoffs, the model equations, and the basic mechanisms of network formation. In Section 3 theoretical and experimental results are reported. The results obtained are discussed in Section 4. Finally, Section 5 reports a detailed technical description of the evolutionary game which is assumed to be the basis of system dynamics.

## 2. Materials and methods

**Figure 1:**
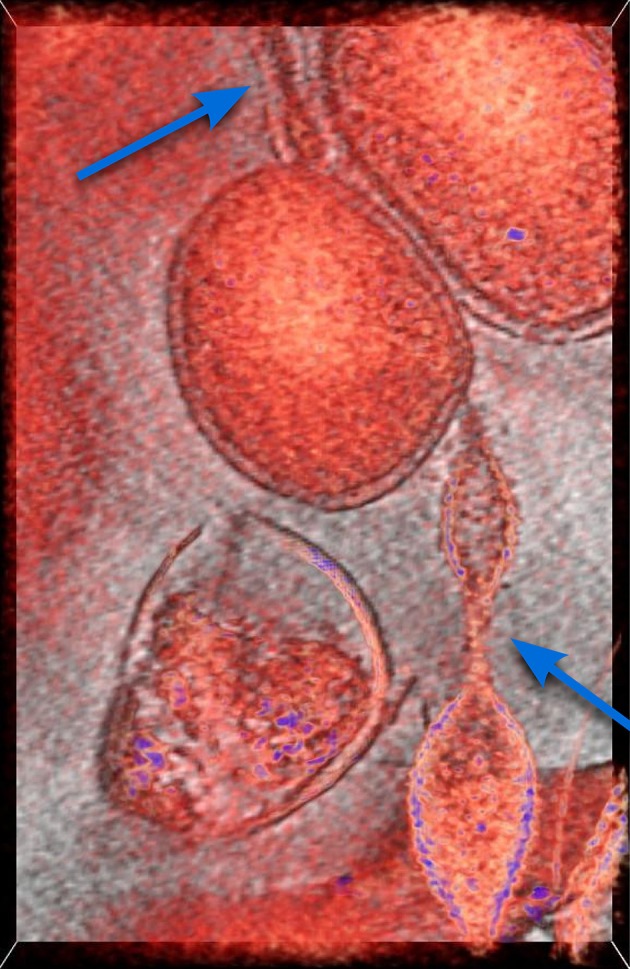
**Cryogenic-electron microscopy image of a small region of a biofilm**. In red, a 50-voxel-thick slice through a tomographic reconstruction overlaid onto a one-voxel-thick slice in gray scale. There is a round cell at the center and what appears to be an extension from a different cell type into it from the bottom, and another at the top. Previous work (see Baker et al., 2010 and references therein) established they belong to different species of archaea. Arrows indicate tubular extensions or appendages connecting the microbial cell in the center to microbial cells of different species. This is established by their typical, clearly different cell walls. For more information see Comolli and Banfield (2014) in this Special Topic.

In this section we introduce the mathematical model describing the mechanism for which the individuals of a population of bacteria are able to make the decision of establishing reciprocal connections aimed at maximizing their probability of survival in a hostile environment. More precisely, cooperation yields the activation of a physical connection between two or more organisms, such as the extensions visible in Figure 1 where the Cryogenic-electron microscopy image of a biofilm revealing the existence of connections among archaea of different species are reported.

### 2.1. Individuals, strategies, decisions, and rewards

We assume that a population of *N* bacteria is located in a spatial domain and that it is composed of two subclasses. The classes differ only for genetic and phenotypic characteristics, and not for their members behavior or decisions. Elements of different classes can create links to exchange genetic material, proteins, metabolic intermediates, etc. The formation of links is assumed to stem from the need to obtain elements allowing bacteria to better resist hostile external conditions (Ben-Jacob et al., 2000). Analogously two elements of the same class are not allowed to create any link. The original conditions are restored once the external situation becomes favorable again and the bacteria are able to reproduce.

In order to be willing to create a link, a microorganism must have enough (finite) available energy to exchange with one or more organisms of another class. The level of energy that bacteria decide to share depends on how averse the environmental conditions are. Moreover, energy transfer is dissipative, because a part of the energy is lost due to distance and effort of linking.

The model developed in this paper assumes that the connections are bilateral; although, one can allow monodirectional links as explained in Section 2.3.

The processes and assumptions described above can be modeled by introducing the following variables:

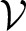
 is the set of all considered bacteria (|
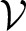
| = *N*);
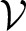
_1_ ⊂ 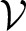 and 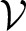
_2_ ⊂ 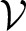 are the subclasses, where 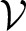 = 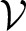
_1_ ∪ 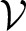
_2_ and 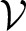
_1_ ∩ 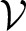
_2_ = ∅;
- ρ_*v,w*_ > 0 is the distance between bacteria *v* and *w*;
- *T_v_* > 0 is the maximum amount of energy that organism *v* can transfer to others.

The decision of an individual to establish a connection with other individuals is modeled using an evolutive game. In the game the players (bacteria) are allowed to choose a strategy in set 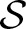. The strategies available to each player consist of the will of being connected to another player. The number of feasible strategies to each player is *N* and we can state a relationship of equivalence between players and strategies. For simplicity of notation, we indicate players and strategies by means of their labels, thus 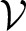 = 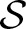 = {1, …, *N*}, and, when needed, players will be labeled by the letters *v*, *w*, … and their strategies by the symbols *s_v_*, *s_w_*, …. For example, if player *v* ∈ 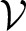 uses strategy *s_v_* ∈ 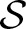 with *v* ≠ *s_v_*, he wishes to connect to player *w* ∈ 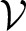, which is such that *w* = *s_v_*. On the contrary, if *v* = *s_v_*, then *v* is connected only to himself. Since self energy transfers are not meaningful, circular connections correspond to the activation of any connection.

Consider two individuals, *v* ∈ 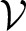
_1_ and *w* ∈ 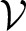
_2_, and suppose that *v* chooses pure strategy *s_v_* ∈ 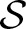 and *w* chooses pure strategy *s_w_* ∈ 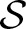. At this point, *v* will receive energy from *w* if and only if *s_v_* = *w* and *s_w_* = *v*. The same holds for *w*. This means that individual *v* is effectively connected to *w* if and only if *w* is also willing to be connected to *v*.

**Figure 2:**
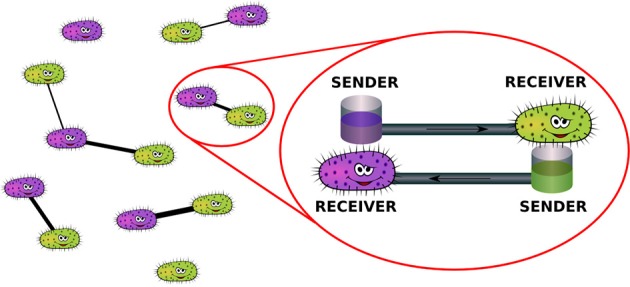
**Mechanism of network formation in the population of bacteria: couples of individuals may decide to reciprocally share a certain amount of energy in order to be able to resist environmental disturbances**.

A pictorial representation of the physical mechanisms described by the model is reported in Figure 2.

The effective energy 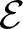
(*w, v*) received by *v* when it is connected to *w* depends on the reciprocal decisions of the two individuals to be connected and on their physical distance. More specifically, energy is defined by:

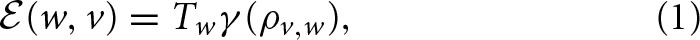



where γ(ρ) is a monotonically decreasing function allowing to quantify the effective available energy after dissipation due to distance. Although multiple specifications are possible, the properties that γ(ρ) should satisfy are the following:

- γ(ρ) ≥ 0 ∀_ρ_ ∈ [0, + ∞);
- γ′(ρ) ≤ 0 ∀_ρ_ ∈ [0, + ∞);
- γ(0) = 1;
- γ(+ ∞) = 0.

Moreover, 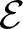
(*w, v*) = 0 if *v* and *w* belong to the same set.

Notice that since *T_v_* is generally different from *T_w_*, 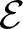
(*w, v*) can be different from 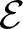
(*v, w*), i.e., the two individuals may earn different rewards from connection.

To develop the model equations describing the biological mechanism of cooperation, we assume that the members of the population are allowed to play suitable games, the strategies of which are the propensity to form connections. The members of the population will be interchangeably called individuals, bacteria or players.

### 2.2. The replicator equation of connections

The state variable of the model is the propensity *x_v,s_v__* of a player to connect to another player. More specifically, the quantity *x_v,s_v__* can be read as a percentage indicating the share of energy that player *v* is available to transfer to player *w*. According to our previous notation, we can also indicate the second player *w* as the strategy that player *v* adopts when he is looking for a connection with him (*w = s_v_*). The distribution of strategies of a single individual *v* of the population is accounted by vector

$$x_{v}=(x_{v,1},\ldots ,x_{v,N}),$$

where

$$\sum_{s_{v}=1}^{N}x_{v,s_{v}}=1\wedge x_{v,s_{v}}\in [0,1]\;\forall v,s_{v}.$$

In general, *x_v,s_v__* ≠ *x_s_v_,v_*, because two individuals starting the process of connection are allowed to independently choose the amount of sharable energy—although both are required to share a minimum energy to make effective connections.

An individual can decide to share his energy with more than one, at which point the vector representing strategy distribution ***x**_v_* may include components strictly less than 1. In this case, it is called *mixed strategy* distribution. On the other hand, solutions with *N* − 1 null and only one unitary component are called *pure strategies* in the underlying game. It is clear to see that mixed strategies include pure strategies.

From a biological point of view pure and mixed strategies indicate that an individual is willing to connect with strictly one or more bacteria, respectively.

The complete distribution of the chosen strategies for the whole population is represented by the variable

$$X=(x_{1},\ldots ,x_{N}),$$

which includes *N*^2^ components, namely *x_v,s_v__*. There are *N* components for each bacterium belonging to each of the two classes 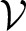
_1_ and 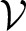
_2_.

The above statements allowed us to define the reward, or payoff, *p^
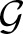
^_v,s_v__*, obtained by player *v* when it connects to *s_v_*, as follows:

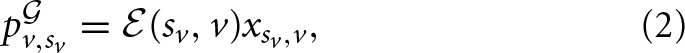



where the superscript 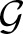 indicates the presence of a graph. Consequently, the average payoff for player *v* over the graph is:

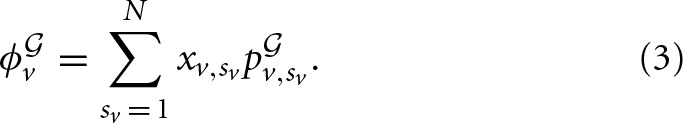



According to the well known theory on evolutionary games (Weibull, 1995; Hofbauer and Sigmund, 1998) and to the same theory extended to networked populations (Madeo and Mocenni, under revision), we can write the *replicator equation* of the game accounting for the graph under construction by bacteria. This equation describes the evolution over time of the distribution of pure/mixed strategy vectors ***x**_v_*, and reads as follows:

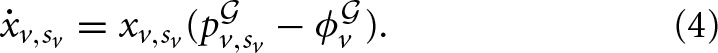



The corresponding Cauchy problem can be obtained from Equation (4) by setting an additional constraint on initial conditions, namely *x_v,s_v__*(*t* = 0) = *x*^0^*_v,s_v__*. It is well known from the theory on ordinary differential equations (ODEs), that this problem has a unique solution, *x_v,s_v__(t)*, *t* ∈ [0, *T*], In the specific system developed here this solution represents the evolution over time of the distribution of the propensities of each bacterium to be connected to any other bacterium present in the systems. Some examples of the evolution of these variables over time are shown in **Figure 6** reported below and in Supplementary Movie 1.

### 2.3. The graph topology

The solution ***X***(*t*) = (***x***_1_, …, ***x**_N_*) of Equation (4) allows us to calculate the *effective connection graph* 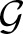
*^E^(t)*, by defining its adjacency matrix ***A**^E^(t)* = {*a^E^_v,w_(t)*}_*v,w*∈
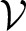
_ as follows:

(5) $$a_{v,w}^{E}(t)=\{\begin{array}{ll} 1 & \text{ if }x_{v,s_{v}}(t)>\eta \wedge x_{w,s_{w}}(t)>\eta \\ 0 & \text{ otherwise} \end{array},$$

where *s_v_* = *w*, *s_w_* = *v* and η ∈ [0, 1] is a given threshold.

The model can also be developed assuming that the effective connection graph 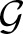
*^E^(t)* is directed, in order to take into account monodirectional connections, arising, for example, when bacterium *v* shares energy with *w*, but *w* does not send anything back. Indeed, one can rewrite graph (5) as follows:

(6) $$a_{v,w}^{E}(t)=\{\begin{array}{ll} 1 & \text{ if }x_{v,s_{v}}(t)>\eta \\ 0 & \text{ otherwise} \end{array},$$

where *s_v_* = *w*. The latter approach goes beyond the scope of the present study, and in the following we only use the bidirectional graph defined by Equation (5).

The parameter η in Equation (5) represents the threshold above which the will to connect becomes an effective link. From a mathematical point of view η is a way of selecting optimal connections, but it also has an interesting biological interpretation because it is correlated to the maximum number of connections that a bacteria is allowed to have. Suppose that bacterium *v* is the most connected in the graph, and let *n_v_* be the number of its connections. Then there are at least *n_v_* components *x_v,s_v__* of vector *x_v_* greater than η. Since the sum of all these components is at most 1, each of them is at least $\frac{1}{n_{v}}$ and hence η < $\frac{1}{n_{v}}$. Thus, η is inversely correlated to the number of connections of the most connected bacterium in the graph.

**Figure 3:**
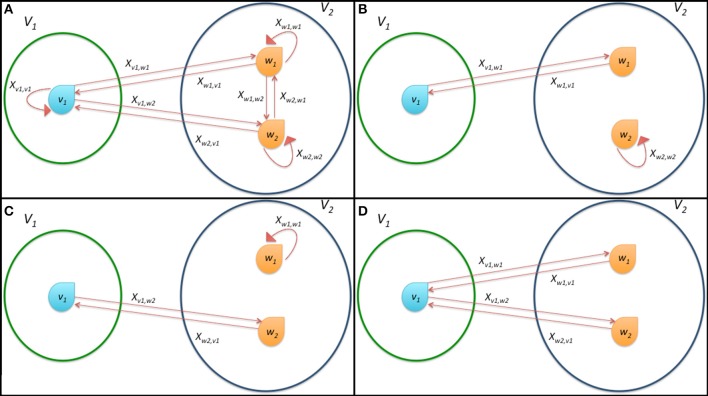
**Schematic representation of the connections among the bacteria in a simple system composed by only three microorganisms divided in the two classes** 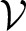
**_1_ and** 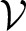
**_2_. (A)** shows all possible connections, **(B,C)** report the effective formation of a single connection and **(D)** represents the onset of multiple connections. The state variables *x_v, w_* describing the dynamics of the system according to Equation (7) are also reported as labels of arcs of the graph.

In order to ease the explanation of the model and the mechanism of link formation we report a schematic representation of possible connections and state variables for a prototypical system composed by the two sets *V*_1_ = {*v*_1_} and *V*_2_ = {*w*_1_, *w*_2_} in Figure 3A. This system is deeply analyzed in the following Subsection 2.3.1.

#### 2.3.1. The system of equations for a simple case

Here we report some theoretical results obtained by developing the mathematical model for a simple example involving *N* = 3 bacteria divided into 2 classes, in particular, 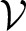
_1_ = {*v*_1_} and 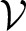
_2_ = {*w*_1_, *w*_2_}), as shown by Figure 3. For the sake of simplicity in the following equations the individuals are simply enumerated from 1 to 3, specifically 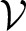
_1_ = {1} and 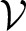
_2_ = {2, 3}. In this case the system is composed of *N*^2^ = 9 ordinary differential equations:

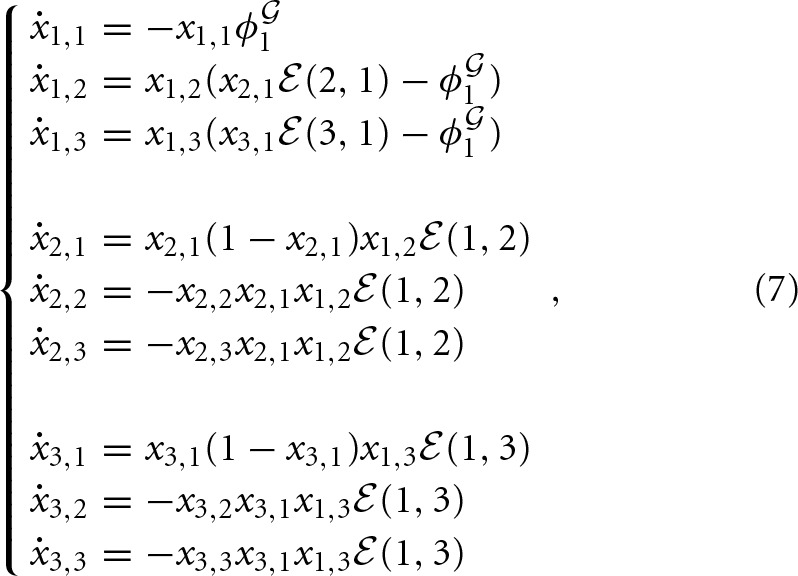



where ϕ^
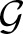
^_1_ = (*x*_1,2_*x*_2, 1_
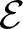
(2, 1) + *x*_1,3_*x*_3,1_
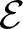
(3, 1)).

As described in Madeo and Mocenni (under revision), pure strategy profiles are always stationary points for system (7). In particular, concerning players 2 and 3, the strategies x^*^_2_ = x^*^_3_ = [1 0 0]^*T*^ are attractive, since *ẋ*_2,1_ ≥ 0, *ẋ*_3,1_ ≥ 0, *ẋ*_2,2_ ≤ 0, *ẋ*_2,3_ ≤ 0 and *ẋ*_3,2_ ≤ 0, *ẋ*_3,3_ ≤ 0 ∀ ***X***. As expected, both players 2 and 3 naturally want to connect to player 1. Moreover, ϕ^
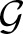
^_1_ ≥ 0 and hence *ẋ*_1,1_ ≤ 0. This means that x^*^_1,1_ = 0 is attractive and x^*^_1,2_ + x^*^_1,3_ = 1. Using these stationary state values, we can rewrite the second and third equations as follows:

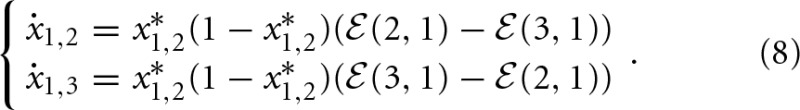



Clearly, x^*^_1_ = [0 1 0]^*T*^ and x^*^_1_ = [0 0 1]^*T*^ are stationary points. These two cases represent situations in which bacterium 1 connects only to bacterium 2 or 3, respectively (see Figures 3B,C). If 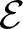
(2, 1) = 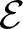
(3, 1), then x^*^_1_ = [0 x^*^_1,2_ 1 − x^*^_1,2_]^*T*^ represents an infinite set of stationary points, with x^*^_1,2_ ∈ (0, 1) and 1 − x^*^_1,2_ ∈ (0, 1). This means that player 1 can potentially establish connections with both players 2 and 3. This happens when x^*^_1,2_ and 1 − x^*^_1,2_ are also greater than η. This result shows that in the particular case in which there are players able to transfer the same amount of energy to a single bacterium, multiple connections are allowed (see Figure 3D).

## 3. Results

In this section we provide some numerical simulation results of the model developed in this paper.

As described in the Materials and Methods Section, the function γ in Equation (1) accounts for the assumption that the mechanism of connection formation depends on the distance between bacteria. The simulations reported in this section have been obtained by using the following specification of γ:

(9) $$\gamma (\rho )=\{\begin{array}{ll} 1-\frac{\rho }{\mu } & \;0\leq \rho \leq \mu \\ 0 & \;\rho >\mu \end{array}.$$

According to Equation (9), in the definition of γ the parameter μ represents a distance threshold for feasible connections; indeed, for each couple of bacteria *v* and *w* that are separated by a distance ρ_*v, w*_ > μ, γ(ρ_*v, w*_) = 0 and hence 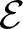
(*w, v*) = 0. In other words, connections over distances greater than μ do not allow any energy exchange and are thus not convenient.

In order to evaluate quantitatively the simulation results we introduce the following indicator of efficiency:

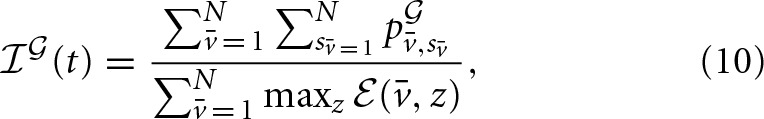



where *v*s are all the connected bacteria and *z* are all bacteria. The indicator evaluates the instantaneous ratio between the total payoff of connected bacteria and the total energy available to bacteria for establishing connections. Notice that the 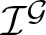 changes over time. The payoff and the number of connected bacteria vary in time.

### 3.1. Experimental results

The results of several numerical experiments developed over a population of 30 bacteria organized into two different classes are reported. The energy available to each bacterium is assigned randomly at the beginning of the simulation.

**Figure 4:**
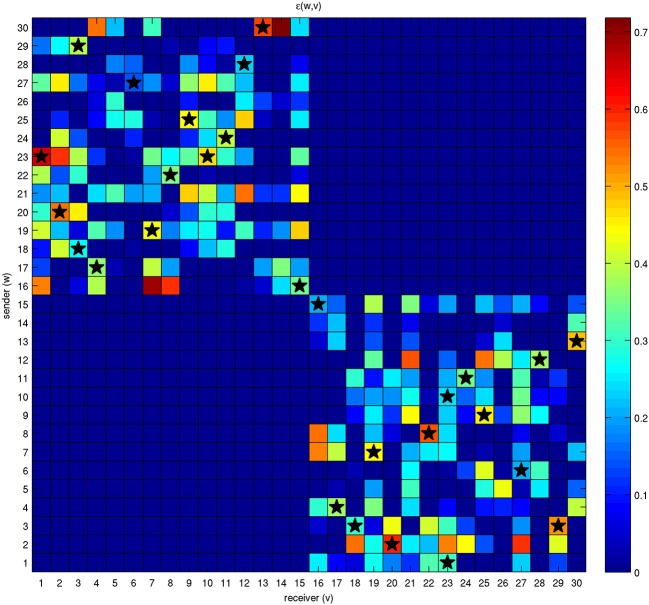
**Energy initially available for exchange among a network of 30 bacteria**. Each bacterium has a quantity of energy available it wants to transfer to the others and it will be allowed to receive an amount of energy from them. These two energies are represented by different colors in the rows and columns of the grid. For example, bacterium 23 wants to share a large amount of energy with bacterium 1; on the contrary, the quantity of energy that bacterium 1 is available to transfer to bacterium 23 is much lower. Nonethelss, the two bacteria will be able to establish an effective connection, although with different intensities, because the corresponding state variables *x*_1,23_ and *x*_23,1_ lie above threshold η. The stars correspond to effective connections and energy exchanges.

The net energy 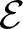 that each bacterium is available to share with other bacteria is initial energy *T* scaled by distance through γ. In other words, this quantity represents the energy that each bacterium is available to send (sender) to another bacterium (receiver). Sender and receiver bacteria are reported as rows and columns of the grid shown in Figure 4, respectively. The stars indicate the presence of a connection among bacteria that has been permanently established according to the mechanisms described by Equation (5).

It is interesting to note that effective connections are possible only when both linked bacteria have enough available energy to share with the other. The antisymmetric parts of the matrix are not involved in the mechanism of energy sharing because the connections are allowed only among individuals belonging to different classes.

**Figure 5:**
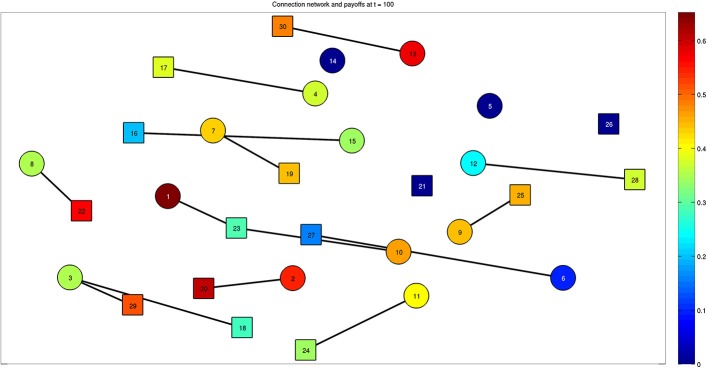
**Final configuration of the network composed of 30 bacteria at steady state (*t* = 100)**. Bacteria belonging to the two classes are represented by different shapes, such as squares and circles. The color associated to each bacterium represents the effective quantity of energy it receives thanks to the established connections. Multiple connections are present, for example bacteria 3 and 23 established connections with bacteria 29–18 and 1–10, respectively. See also Supplementary Movie 1, which reports the whole dynamics and provides the connection between Figures 4 and 5.

When the steady state is reached, we find the presence of effective stable connections reported in Figure 5 for a run with 30 bacteria over 100 time steps. The colors of the nodes correspond to the value of the payoff described by Equation (2), calculated at the steady state. With parameter values η = 0.2 and μ = 0.2, we can see that some bacteria are able to activate multiple connections. Connections involving more than 3 bacteria are also possible for different values of the parameters, as reported by the following figures. The interplay between Figures 4 and 5 is shown by Supplementary Movie 1, where the dynamics is reproduced, and the onset of links can be followed over time (left inset of the movie). The inset on the right part of the movie reports the state variable *x_v, w_* for each bacterium.

**Figure 6:**
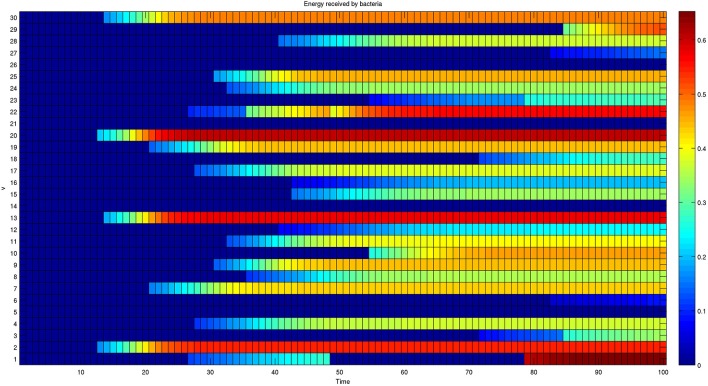
**Dynamics of the energy received by each member of a network composed of 30 bacteria**. The colors reported in each row represent the energy received by a single bacterium over time. The energy received initially is null for all bacteria. The presence of oscillating behaviors, such as the one shown by bacterium 1, shows that the final configuration is also the result of transient activated/deactivated connections.

Furthermore, the complete dynamics of the energy received by each bacterium is reported in Figure 6.

**Figure 7:**
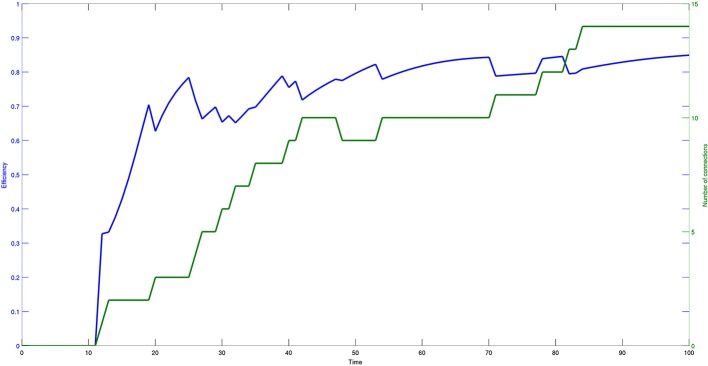
**Time course of efficiency (blue) and number of effective connections (green) of a network composed of 30 bacteria**. The data are plotted with different scales and the model parameters are set as follows: η = 0.2 and μ = 0.2.

Figure 7 shows the values of the efficiency indicator (10) and the number of connections arising in the evolutive process. Both efficiency and number of connections have a monotonically increasing and saturating dynamics, showing that the process reaches a steady state quite fast.

**Figure 8:**
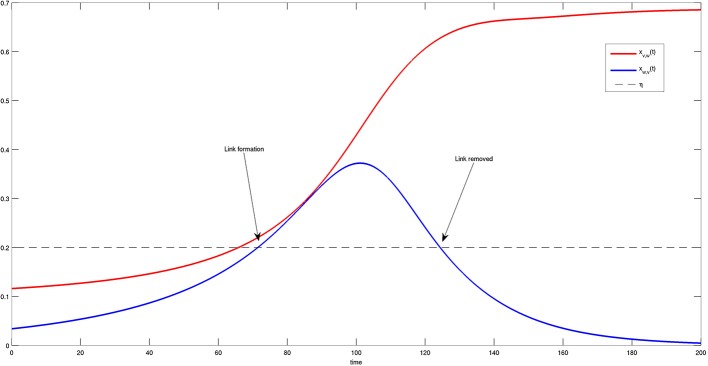
**Time course of two components *x_v, w_(t)* (red) and *x_w, v_(t)* (blue) of the system state *X*(t)**. When both components are greater than η (dashed black line), a link between *v* and *w* is formed. The link disappears when component *x_v, w_(t)* falls below the threshold η. This transient behavior is in agreement with the results shown in Figure 6.

More details on the state variables and solutions of Equation (4) are provided in Figure 8. In particular, the time course of the dynamics of two bacteria is reported and compared to threshold η. One can observe that the first link between the two bacteria is formed at approximately *t* = 70, when both variables exceed threshold η. Nevertheless, the connection is removed later on at approximately *t* = 125, when one of the two components falls below the threshold. It is interesting to note that the bacteria try to activate several connections before they are able to reach a steady state, i.e., a stable and permanent configuration.

**Figure 9:**
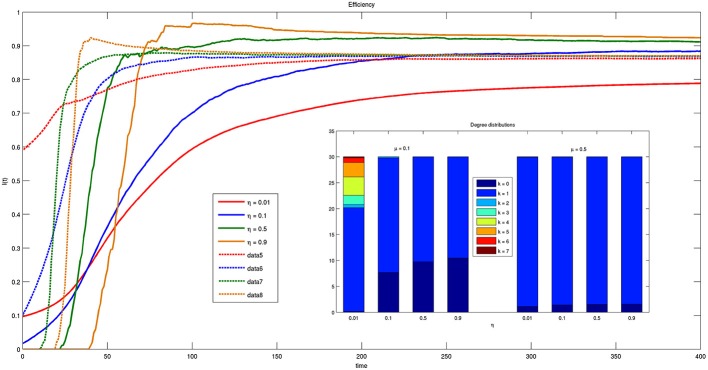
**Time course of efficiency for different values of threshold η = {0.01, 0.1, 0.5, 0.9} and parameter μ = {0.1, 0.5}**. Solid lines correspond to μ = 0.1 and dashed lines to μ = 0.5. The inset reports the histogram of connections for μ = 0.1 and μ = 0.5 at *t* = 200.

Figure 9 shows the time course of the efficiency indicator on a time interval of 400 time instants for different values of parameter η. The increase of efficiency over time indicates that the system is moving toward a more efficient state. Moreover, for small values of μ (solid lines) efficiency is higher for larger ηs, while for large values of μ (dashed lines) the asymptotic values reached by efficiency are independent of η. Recall that μ is related to the amplitude of the spatial region to which each bacterium is allowed to look for connections with others. The independence of the final configuration of the network on parameter η spontaneously resulting from the model is very important because there are presently no guidelines for choosing appropriate values of the threshold η.

Another significant result emerging from the inspection of Figure 9 is that for small μs values of η exist for which efficiency increases more quickly. In particular, for η = 0.5, the asymptotic stable value of efficiency is reached much faster than for any other value.

The inset of Figure 9 reports the histogram of the different kinds of connections that can be established by bacteria with respect to η and for different μ. As one can see, for small μ multiple connections are feasible while for high μs only one to one connections are effectively possible. As mentioned system efficiency reduces for small values of μ the system efficiency reduces. This result confirms that single connections are more efficient and robust. One should also consider that in these present simulations we assume to have only two classes of bacteria, and multiple connections would involve more that one bacterium belonging to the same class, i.e., providing the same information content. In this sense, it seems obvious that single connections are more efficient than multiple ones. Future works will be devoted to compare the solution provided by the proposed model with optimal connection networks.

**Figure 10:**
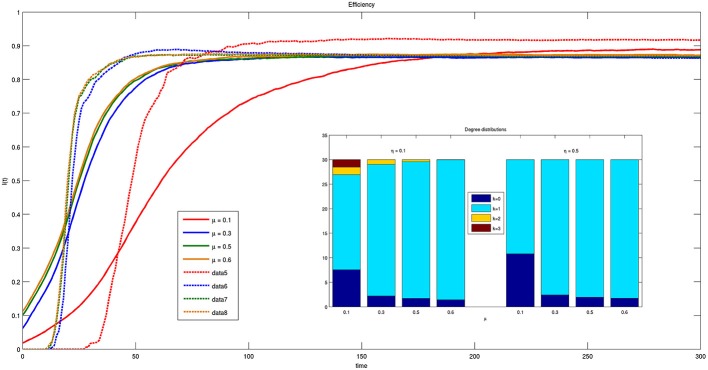
**Time course of efficiency for different values of parameter μ = {0.1, 0.3, 0.5, 0.6} and threshold η = {0.1, 0.5}**. Solid lines correspond to η = 0.1 and dashed lines to η = 0.5. The inset reports the histogram of connections for η = 0.1 and η = 0.5 at *t* = 300.

In Figure 10 the values of efficiency are reported as a function of time for different values of parameter μ. Notice that in this case both the efficiency and its derivative are independent on parameter μ except for μ = 0.1. The curves have the same dynamics for μ = {0.3, 0.5, 0.6}, while for μ = 0.1 the system takes more time to reach steady state. This mechanism is even stronger for smaller ηs, reported as solid lines in the figure. Moreover, in the latter case, the inset of Figure 10 shows that for small η bacteria are allowed to establish multiple connections with approximately the same efficiency.

**Figure 11:**
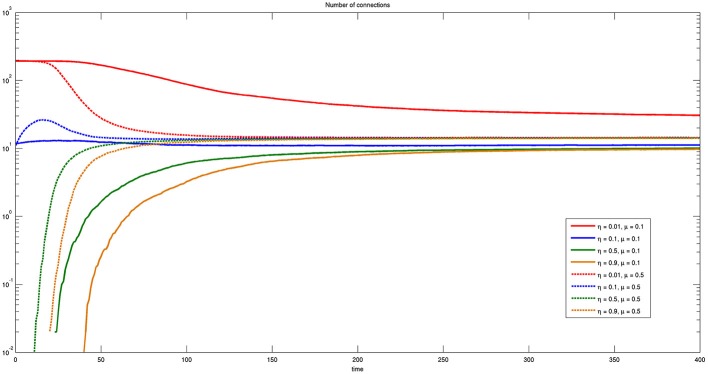
**Time course of the number of connections for different values of η and μ**. Here η = {0.01, 0.1, 0.5, 0.9} and μ = {0.1, 0.5}. Some connections are present at the initial time when the threshold η is sufficiently low.

Figure 11 reports the time course of the number of connections for different values of η and μ, where solid lines correspond to small and dashed lines to large values of μ, respectively. It is worthwhile to note that the number of connections is independent on both parameters η and μ except for the case of η = 0.01 and μ = 0.1. In this case the number of connections is higher, even though efficiency is lower, shown by the solid red line of Figure 9. A small value of η means that almost any connection is possible and a small value of μ means that the spatial horizon where the bacteria are allowed to look for connections is also very small. Thus, the bacteria will be only allowed to establish multiple one to one short range connections and probably lose the more efficient ones.

Notice that for small values of η some connections already exist at *t* = 0. In fact, the initial values of the state variables assigned randomly to some couples of bacteria may fall below the threshold from the beginning.

**Figure 12:**
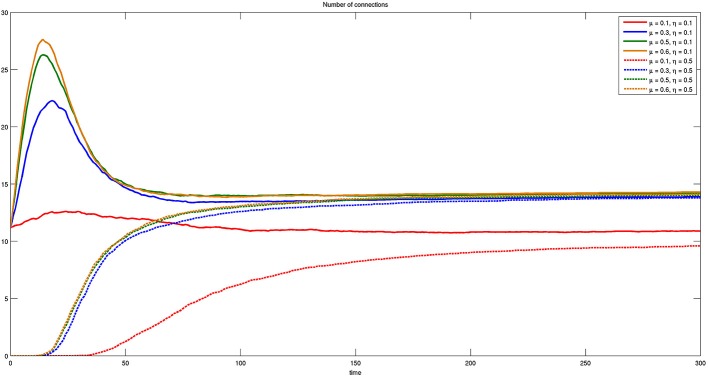
**Time course of the number of connections for different values of η and μ. Here η = {0.1, 0.5} and μ = {0.1, 0.3, 0.5, 0.6}**. The presence of initial connections and transient behavior are evident for η = 0.1.

In Figure 12 we report the same results of Figure 11, but with respect to parameter μ. The most important thing to notice in this case is that the number of connections for small μ and any η is less than any other parameter value. A further significant result is the transient behavior occurring for η = 0.1 and μ = {0.3, 0.5, 0.6}, where groups of bacteria experiment with connections after which they can decide to regress.

## 4. Discussion

This paper is the first attempt to develop a mathematical model describing the activation of suitable physical connections among two or more bacteria experiencing hostile environmental conditions. Contrary to standard ways of interpreting collaboration and clustering among groups or subgroups of bacteria, the model proposed in this study describes a mechanism based on the strategies of individual players. Instead of interpreting patterns as a result of macroscopic and collective behavior involving a large part of the population of bacteria, our model allows two or more bacteria to make the decision of connecting to another specific bacterium of a different species.

An important result of this work is that bacteria (microorganisms) prefer one to one rather than multiple connections because the first ones are more efficient, optimal and robust. This fact reproduces what has been experimentally observed in biofilms, where interspecies interactions do not produce a “common good” such as the constituents of biofilm architecture, but all associations are cell-to-cell (see Comolli and Banfield, 2014). In our framework, these connections result from spontaneous co-evolutive dynamics of groups of bacteria, and are the natural consequence of the basic assumptions under evolutionary game theory. Thus, the fact that we did not need to include any additional a priori assumption or information in the model to obtain the above results suggests our model captures a fundamental aspect of microbial life within biofilms.

When environmental conditions are very averse, and microorganisms need the recruitment of diversified biological components such as metabolites, proteins, defense mechanisms, nucleic acids, etc. (see Mitri et al., 2011), we may suppose that each component is provided by a bacterium belonging to a particular class. At this point, we expect that multiple connections will be more efficient than one-to-one. The present work can be powerfully extended to account for the presence of more than two subclasses of bacteria, and hence more than two kinds of energies available for exchanges.

An additional significant result of the paper is that the asymptotic values of efficiency at the global level, and number of connections are almost independent of the connection activating threshold η and distance threshold μ. Recall that two bacteria belonging to different classes establish a link when they are both willing to connect, i.e., their state variables are greater than parameter η. The connection mechanism dissipates a certain amount of energy and allows the system to reach a certain level of efficiency. On the other hand, parameter μ mostly influences the total number of connections, even though at steady state the same level of efficiency is reached. Put together, the above results indicate that the model has a globally attractive steady state, which is optimum with respect to the strategies' distribution of the underlying game. Nevertheless, parameter η significantly influences how fast the above steady state is reached during the first phases of the process of network formation.

The recruitment of the optimal network configuration needs deeper investigation. For example, introducing suitable functions to be optimized on the basis of the techniques presented in the field of graph theory. This will substantially help the understanding of still unknown biological mechanisms of structure formation associated with cell-cell interfaces, such the ones described in Comolli and Banfield (2014) for *arcahea*.

In conclusion, our results show that since the environmental conditions constantly change microorganisms are responding not just to the changes in external factors but in concomitance with the changes adopted by the entire system of microbes. The network thus provides a mechanism of resilience and robustness. In addition, the network of connections we show, linking organisms across a dynamic range of physical connections, should provide the basis for linked evolutionary changes under pressure from a changing environment.

## Author contributions

Luis R. Comolli, Dario Madeo, and Chiara Mocenni contributed to the original motivation, ideas, and exploration of modeling strategies for the study. Dario Madeo and Chiara Mocenni worked on conceptualization, development and analysis of the mathematical model, and performed numerical simulations. Luis R. Comolli, Dario Madeo, and Chiara Mocenni analyzed the results and their conceptualization. Luis R. Comolli, Dario Madeo, and Chiara Mocenni contributed equally to the writing of the manuscript.

## Funding

The study is partially funded by the project “Evolutionary Games on Networks for Modeling Complex Biological and Socio-Economic Phenomena” financed by CNPq (Program Science without Borders, N. 401795/2013-6).

### Conflict of interest statement

The authors declare that the research was conducted in the absence of any commercial or financial relationships that could be construed as a potential conflict of interest.

## 5. Appendix

The details of the game underlying the model presented in the Materials and Methods Section are provided. In particular, the strategies of the game correspond to the willingness of the players to become connected to one or more other players. For this reason, the payoffs of the game are calculated on the basis of the energy that each player is allowed to share. Thereupon, the payoffs as well as energy are functions of time.

### 5.1. Calculating the payoffs of the game

According to the approach proposed in Madeo and Mocenni (under revision), we need a matricial definition of payoff functions *p^^_v,s_v__* and ϕ^^_*v*_ in order to describe the model using the replicator equation on a networked population. To this aim, we define the payoff obtained by *v* when he plays with *w* and strategies used are *s_v_* and *s_w_*, respectively:

In other words, player *v* will earn a payoff (energy) when playing with player *w* if and only if both *v* and *w* decide to connect to each other(i.e., *s_v_* = *w* and *s_w_* = *v*). The total payoff (energy) obtained by player *v* ∈ _*i*_ is defined as the sum of all energy obtained from players *w* ∉ _*i*_. Namely:

In the model developed in this paper, pure strategies are chosen by players that are willing to connect to only one other player, while mixed strategies are such that eventually an individual will share its energy with more than one individual. Notice that in this case sharing all the available energy with only one player corresponds to adoption of a pure strategy. The pure strategy *s_v_* of player *v* can be described by the unitary vector ***e**_s_v__* = (0, …, 1, …, 0), where 1 is placed at the *s_v_*-th position. Using Equation (A11), we can define the payoff matrix for each couple of bacteria *v* and *w*:

(A13) $$B^{v,w}={\left\{b_{s,r}^{v,w}\right\}}_{s,r=1,\ldots ,N}.$$

Thereafter, we can make use of Equation (A13) to rewrite Equation (A12) in a matricial fashion:

where ***e**^T^_s_v__**B**^v,w^**e**_s_w__* = *b^v,w^_s_v_,s_w__*.

Mixed strategies describe how player *v* distributes his energy. This distribution is described by vector *x_v_* = (*x*_*v*,1_, …, *x_v,N_*), where *x_v,s_v__* ∈ [0, 1] and $\sum_{s_{v}=1}^{N}x_{v,s_{v}}=1$. Practically, *x_v,s_v__* represents the percentage of *T_v_* that *v* wants to share with *s_v_* ≠ *v*. *x_v,s_v__* can be also read as the propensity of *v* to create a link with a player *w* = *s_v_*. *x_v,s_v__* ∈ [0, 1], for which *v* = *s_v_*, is the fraction of energy that *v* does not share. In this case *x_v, v_* can also be read as the propensity of *v* to remain unlinked. The previous notation allows us to calculate the payoff, hereafter indicated by *p^^_v,s_v__*, that player *v* obtains when he decides to use pure strategy *s_v_* against a player that uses mixed strategy ***x**_w_*:

where the superscript  indicates the presence of a graph of possible connections. The model developed in this paper will be mainly used to recover the graph topology.

The average payoff obtained by player *v* when it plays a mixed strategy ***x**_v_* is:
